# Esophageal squamous cell carcinoma in a patient with *BRCA1* mutation: a rare association

**DOI:** 10.3332/ecancer.2024.1730

**Published:** 2024-07-16

**Authors:** Mohammad Saad Salim Naviwala, Mirza Rameez Samar, Daania Shoaib, Fizza Akbar, Romana Idrees, Yasmin Abdul Rashid

**Affiliations:** 1 Department of Medical Oncology, Aga Khan University Hospital, Karachi 74800, Pakistan; 2 Department of Women and Child Health, Aga Khan University Hospital, Karachi 74800, Pakistan; 3 Department of Pathology, Aga Khan University Hospital, Karachi 74800, Pakistan

**Keywords:** squamous cell carcinoma, next-generation sequencing, BRCA1, esophageal neoplasms

## Abstract

**Background:**

Esophageal neoplasms rank as the 7th most common cancers in the world. Squamous cell carcinomas of esophagus (SCCE) are the predominant subset, linked to a number of genetic alterations. Gene-driven tumour pathways are being increasingly identified with the emerging role of next-generation sequencing.

**Case presentation:**

We report a case of an 82-year-old male patient who was diagnosed with SCCE involving the cervical region. He received definitive concurrent chemoradiotherapy with Carboplatin and Paclitaxel. To trace the family history of malignancy, a genetic test was carried out which turned out to be a pathogenic BRCA1 variant.

**Conclusion:**

SCCE arising in the context of known *BRCA1* mutation has been rarely reported to date. Testing for these mutations should be considered in patients who present with esophageal cancer, especially in the backdrop of familial neoplasms.

## Background

Squamous cell carcinoma of esophagus (SCCE) is an aggressive neoplasm that is associated with a high relapse rate and mortality rate [1]. It has a complex carcinogenic mechanism that has been related to poor prognosis. Recent studies have demonstrated several critical genes and pathways crucial to tumorigenesis of esophageal cancer. The use of next-generation sequencing (NGS) platforms has played a pivotal role in providing comprehensive methods to detect somatic and germline genome alterations in cancers. Detection of disease-causing, pathogenic (P), or likely pathogenic (LP) variants (mutations) through the use of NGS is increasing which has improved our understanding of cancer biology. Researchers have exploited this technology to better understand the tumorigenesis of SCCE which is leading to finding several critical genes and pathways that provide drug targets to guide future precision medicine in oncology. In addition to the environmental risk factors associated with SCCE, there is growing evidence that supports the involvement of genetic and epigenetic risk factors in the tumorigenesis of ESCC.

Extensive work, enabled by NGS platforms; is being carried out; to understand the somatic genomic alterations in ESCC. Somatic variants attributing to tumorigenesis and having a possible disease prognostic value had been identified in genes including, *TP53*, *EP300*, *NFE2L2, CSMD3, CCND1, CDKN2A, CREBBP, RB1, KMT2D, KMT2C, KDM6A, FAT1, FAT2, FAT3, FAT4, AJUBA, NOTCH1, NOTCH2, NOTCH3, FBXW7, PTCH1 and PIK3CA* [2–4] Additionally, limited published work has however shown germline variants in *TP53, BRCA1, BRCA2* and* RECQL4* contributing to familial cases of ESCC [5, 6].

Of note, the most widely studied genes are two tumour suppressor genes, *BRCA1* and *BRCA2,* both of which influence homologous recombination in DNA repair pathway [7]. Unquestionably, P and LP or disease-causing variants of these genes, increase the risk of malignancies, most notably breast and ovarian cancers. Apart from these, they are also associated with an increase in the risk of pancreatic cancers, prostatic cancers and male breast cancers [8]. With the development and exploration of more cost-effective gene sequencing methods, both somatic and germline and mutations in *BRCA* genes have been detected in several cancers. *Testing for BRCA1/2 has exponentially increased as part of the patient’s clinical management and eligibility for* targeted therapeutic agents with the introduction of poly-ADP ribose polymerase inhibitors in cancers with *BRCA* mutations [9]. In a family-based association review, the relative risk (RR) was found to be (*RR 2.9, 95% CI 1.1–6*) in esophageal and *(RR 2.4, CI 1.2–4.3*) in gastric cancer in families with known *BRCA1* mutations [10]. Another study showed the cumulative lifetime risk of developing esophageal cancers in BRCA1/2 carriers by the age of 85 years at 5.2% (95% CI, 1.7%–8.5% [11].

Here, we report a patient with confirmed germline *BRCA1* mutated SCCE.

## Case report

An 82-year-old gentleman, with no known prior co-morbidities, and an Eastern Cooperative Oncology Group Performance Status score of 1, presented to our institution with complaints of difficulty swallowing for 3 months. He narrated that it started off with solids and gradually progressed to liquids as well. Along with this, he also reported an unintentional weight loss of around 4 kg in this interim. He did not report any history of substance abuse or past addictions.

**Figure 1.**
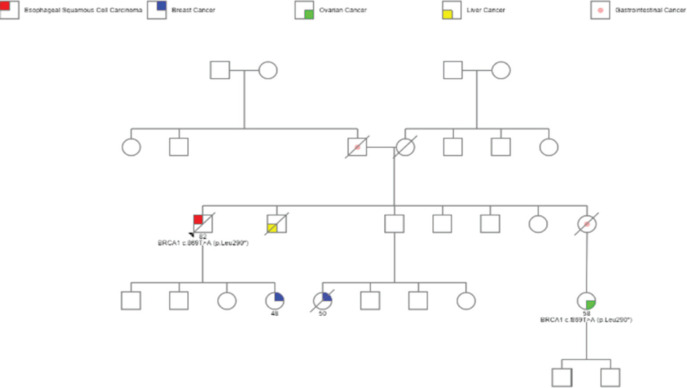
Flowchart showing patient’s pedigree information of malignancies, which includes patient’s diagnosis of SCCE, gastrointestinal and liver cancers in siblings, breast cancer in daughter and breast and ovarian cancers in nieces.

However, further questioning highlighted a significant family history of malignancies. Three first-degree relatives (sister, brother and daughter) had been diagnosed with gastrointestinal malignancies and another daughter had been diagnosed with breast carcinoma, illustrated in Figure 1.

On general physical examination, he was of average build and all vital parameters were within reference ranges. Systemic examination was found to be unremarkable. Initial relevant blood investigations had been performed at a different healthcare facility which were all within the reference ranges. A Barium Swallow had revealed a short segment of irregularity and mucosal destruction with multiple filling defects at the lower cervical and upper dorsal esophagus (C7-T1 level). This was correspondent on a contrast-enhanced computed tomography scan of the neck and chest, which demonstrated an 6 cm upper esophageal thickening causing luminal narrowing.

**Figure 2.**
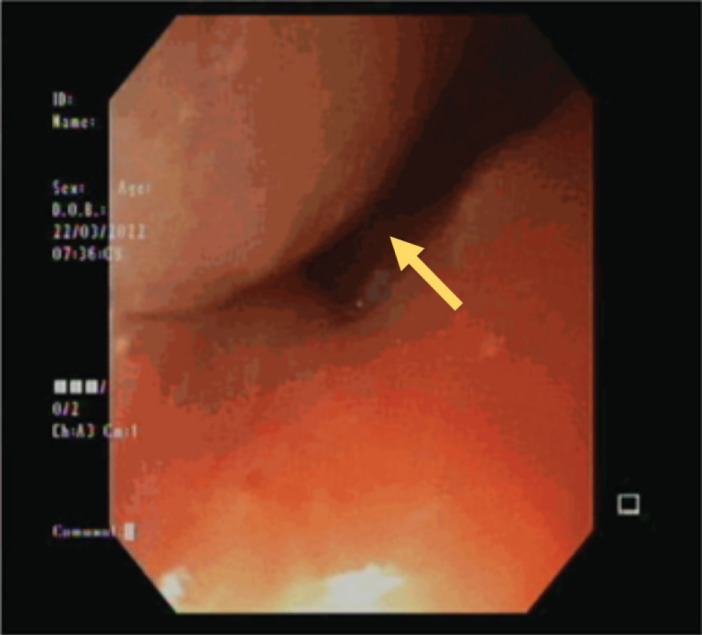
Endoscopic visualisation of the esophageal mass.

**Figure 3.**
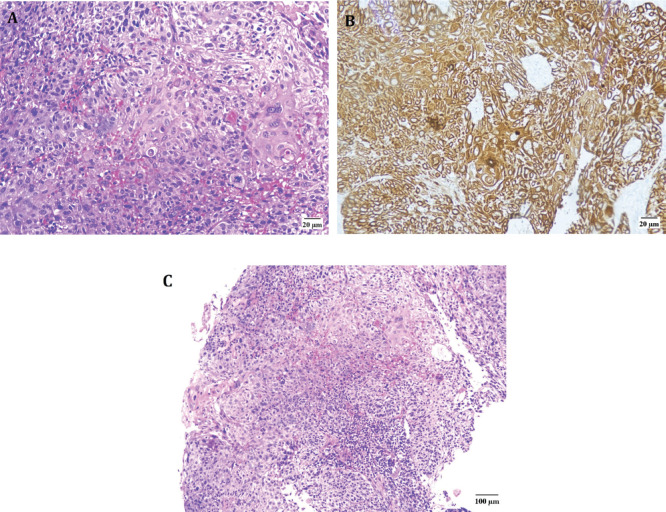
Microscopic findings of the upper esophageal mass. (a): High-power view (40x) displaying squamous cell carcinoma with areas of keratinization. (b): High-power view (40x) showing positive CK5/6 immunohistochemical stain in neoplastic cells. (c): Low-power image (10x) revealing squamous cell carcinoma.

He was advised and subsequently underwent an Esophagogastroduodenoscopy, where an upper esophageal mass was visualised just below the upper esophageal sphincter (Figure 2). Biopsy of this mass was performed and histopathology was consistent with moderately differentiated keratinizing squamous cell carcinoma (Figure 3).

**Figure 4.**
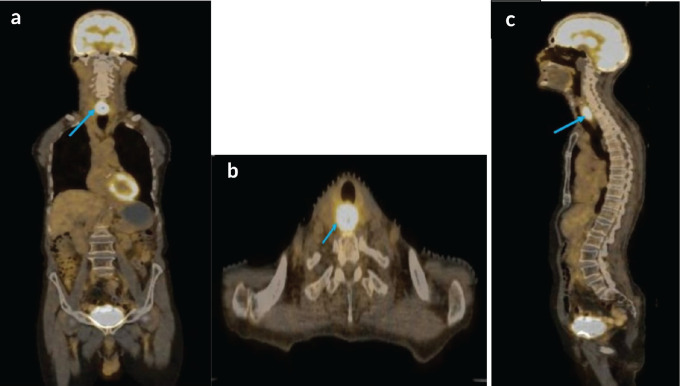
(a–c): Coronal axial and sagittal views respectively, of staging PET-CT scan showing FDG avid, cervical esophageal mass, with normal uptake seen over brain.

A staging positron emission tomography with computed tomography (PET-CT) scan was performed which demonstrated a circumferential thickening of the cervical esophagus, 2.8 × 1.9 × 4 cm in size with a standardised uptake value (SUV_max_) of 14 (Figure 4). This was seen abutting neighboring structures without any invasion. No regional or distant metastases were noted.

Definitive concurrent chemotherapy and radiation, i.e., combined modality treatment, were recommended for the patient. Before initiating therapy, he underwent percutaneous endoscopic gastrostomy tube placement. He received weekly Carboplatin (Area under the curve-2) and Paclitaxel (50 mg/m^2^) alongside 30 fractions of concurrent radiation therapy over the course of 5 weeks, which he tolerated without incident. However, tragically, the patient’s condition deteriorated rapidly, and he passed away before undergoing the end-of-treatment scans to assess his response to therapy. The cause of death was attributed to septic shock secondary to a severe pulmonary infection, highlighting the challenges in managing advanced-stage esophageal squamous cell carcinoma, particularly in elderly patients.

In view of his strong family history of multiple malignancies, he was referred to the Hereditary Cancer Clinic. After a detailed pre-test counselling session, a Multi-Cancer Genetic Panel (84 genes) at an outsourced commercial genetics laboratory (Invitae Genetics, US) was sent. He harbored a P variant in *BRCA1* and additionally, two variants of uncertain significance (VUS) in *ATM* and *CDH1*, latter of which is unlikely to be contributing to disease, as they were present in the population database (gnomAD) and did not meet the criteria to be classified as P/LP variant. The variant classification is based on the American College of Medical Genetics and Genomics criteria. The P variant identified is *BRCA1*, Exon 10, c.869T>A (p. Leu290*). The variant creates a premature translational stop signal, leading to disruption or absence of protein product. The resultant loss-of-function variant in *BRCA1* accelerates disease causation, this variant is absent from the population database (gnomAD) but have been found in the clinical database (ClinVar: 371981).

## Discussion

Esophageal cancer is classified among the most common malignancies, as it ranked the 7th most frequent neoplasm in its occurrence and the 6th most significant cancer leading to mortality in 2020s [12]. Histologically, the two subgroups of esophageal cancer widely known are adenocarcinoma (AC) and squamous cell carcinoma. Between the two, squamous cell carcinoma is the leading cause, making up to more than 85% of the global burden among all esophageal cancer cases [13].

SCCE is prevalent in Northern Iran, Central Asia and North-Central China in contrast to AC, which is predominantly found in highly developed regions such as Europe and North America. Even in Pakistan, SCCE remains the dominant histology, with most cases found in Baluchistan [14]. These subtypes are highly influenced by a variety of risk factors. Smoking and alcohol use, diets rich in nitrosamines and polycyclic aromatic hydrocarbons, and human papillomavirus increase risk of SCCE whereas the major risk factors for AC include chronic gastro-esophageal reflux disease, Barrett’s esophagus, obesity, diets rich in saturated fat and red meat.

For patients who present with locally advanced esophageal cancer, the gold standard treatment is tri-modality i.e. the combination of neo-adjuvant chemoradiotherapy followed by surgery, based on the results of CROSS trial which showed median overall survival twice to that seen with surgery alone [15]. Kamarajah *et al* [16] reported superior survival rates in esophageal cancer with tri-modality than neo-adjuvant chemotherapy followed by surgery. Tri-modality not only improves the pathological complete response rate but also increases rate of R0 resection as reported by Yang *et al* [17].

The understanding of genomic impact on immune landscape in the modern era, have translated into emerging utilisation of NGS as a tool for multi-gene panel analysis. These genetic variants can lead to benign or malignant changes in the protein expression or function. Sawada *et al* [18] reported 15 distinct genes involved in the pathogenesis of SCCE with *TP53* being the most common. Among the most widely studied are *BRCA* genes, which are involved in repair pathways of DNA duplex and are commonly associated with neoplasms involving breast, ovaries, prostate, pancreas, colo-rectum and stomach. Between the two commonly known *BRCA* genes, *BRCA2* harbors a greater risk of gastrointestinal cancers, in particular colorectal cancers [19]. In addition to the BRCA1 mutation, the patient’s genetic testing revealed a VUS in CDH1. CDH1 or E-cadherin, is a tumour suppressor gene associated with various cancers. It regulates cell adhesion and proliferation, and its dysregulation can contribute to tumour progression and metastasis [20]. While the significance of the CDH1 variant in this case is unclear, its involvement underscores the importance of further investigation.

Little has been known regarding the association of *BRCA* genes to esophageal cancers. Only a few studies have revealed the association of loss of function mutation in *BRCA2* with familial esophageal squamous cell carcinomas [21]. Liang *et al* [6] also indicated the potential role of *BRCA2* in familial esophageal squamous cell carcinomas. Recent literature has also shown the activity of platinum-based regimens in *BRCA*-mutated SCCE [22].

Our case had SCCE with an underlying *BRCA1* mutation in the background of a significant family history for malignancies. While a comprehensive germline multi-cancer panel comprising of 84 genes, did not show any other P/LP variants in the patient, genomic alternations at the somatic level in the tumour were not investigated. As a result of comprehensive germline analysis, it seems that germline *BRCA1* mutation is a driver mutation however further work is warranted to better elucidate this.

The presence of a BRCA1 mutation in this case suggests potential implications for treatment strategies. BRCA-mutated tumours, including SCCE, may exhibit heightened sensitivity to platinum-based chemotherapy regimens due to DNA repair defects. Consequently, incorporating platinum-based agents like carboplatin into the treatment regimen could enhance therapeutic outcomes. However, further research and clinical trials are necessary to validate the efficacy of platinum-based chemotherapy in BRCA1-mutated SCCE.

Overall, our findings suggest a possible association between BRCA1 and SCCE, hinting at the prospect of targeted therapies. Additionally, our study underscores the importance of tailored surveillance for associated malignancies and the identification of at-risk family members.

## Conclusion

Esophageal cancers can be uncommonly driven by an underlying *BRCA* mutation. Patients who have a significant history of familial malignancies should be considered for genetic testing for timely identification of these P variants.

## List of abbreviations

AC: Adenocarcinoma; LP: Likely pathogenic; NGS: Next generation sequencing; P: Pathogenic; SCCE: Squamous cell carcinoma of esophagus; VUS: Variant of uncertain significance.

## Conflicts of interest

The authors declare that they have no competing interests.

## Funding

No specific funding has been used for manuscript writing or reporting.

## Consent for publication

Written informed consent was obtained from the patient for the publication of this case report and any accompanying images. A copy of the written consent is available for review by the Editor-in-Chief of this journal.

## Ethics approval and consent to participate

Not applicable.

## Availability of data and materials

The datasets used and/or analysed during the current study are available from the corresponding author on reasonable request.

## Author contributions

SN, MRS, DS gave a relevant contribution in writing the original manuscript. SN also contributed to the conception, formatting and revision of the final manuscript. FA was involved in reviewing and providing critically important content for the manuscript. YAR and RI reviewed the final version of the manuscript. All authors read and gave their approval for the final version to be published.

## References

[1] Starr J, Ramnaraign B (2020). Germline BRCA1 mutated esophageal squamous cell carcinoma. Rare Tumors.

[2] Deng J, Chen H, Zhou D (2017). Comparative genomic analysis of esophageal squamous cell carcinoma between Asian and Caucasian patient populations. Nat Commun.

[3] Gao YB, Chen ZL, Li JG (2014). Genetic landscape of esophageal squamous cell carcinoma. Nat Genet.

[4] Munari FF, Dos Santos W, Evangelista AF (2021). Profile of esophageal squamous cell carcinoma mutations in Brazilian patients. Sci Rep.

[5] Deng J, Weng X, Ye J (2019). Identification of the germline mutation profile in esophageal squamous cell carcinoma by whole exome sequencing. Front Genet.

[6] Liang Z, Hu W, Li S (2020). Germline BRCA2 truncating mutation in familial esophageal squamous cell carcinoma: a case controlled study in China. Med Sci Monit.

[7] Powell SN, Kachnic LA (2003). Roles of BRCA1 and BRCA2 in homologous recombination, DNA replication fidelity and the cellular response to ionizing radiation. Oncogene.

[8] Akbar F, Siddiqui Z, Waheed MT (2022). Spectrum of germline pathogenic variants using a targeted next generation sequencing panel and genotype-phenotype correlations in patients with suspected hereditary breast cancer at an academic medical centre in Pakistan. Hered Cancer Clin Pract.

[9] Zimmer K, Kocher F, Puccini A (2021). Targeting BRCA and DNA damage repair genes in GI cancers: pathophysiology and clinical perspectives. Front Oncol.

[10] Moran A, O'Hara C, Khan S (2012). Risk of cancer other than breast or ovarian in individuals with BRCA1 and BRCA2 mutations. Fam Cancer.

[11] West HJ, Jin JO (2015). JAMA oncology patient page. Performance status in patients with cancer. JAMA Oncol.

[12] Wang ZX, Cui C, Yao J (2022). Toripalimab plus chemotherapy in treatment-naïve, advanced esophageal squamous cell carcinoma (JUPITER-06): a multi-center phase 3 trial. Cancer Cell.

[13] Arnold M, Laversanne M, Brown LM (2017). Predicting the future burden of esophageal cancer by histological subtype: international trends in incidence up to 2030. Am J Gastroenterol.

[14] Asghar MS, Khan NA, Kazmi SJH (2021). Clinical, epidemiological, and diagnostic characteristics of esophageal carcinoma in a Pakistani population. Ann Saudi Med.

[15] Shapiro J, Lanschot JJB, Hulshof M (2015). Neoadjuvant chemoradiotherapy plus surgery versus surgery alone for oesophageal or junctional cancer (CROSS): long-term results of a randomised controlled trial. Lancet Oncol.

[16] Kamarajah SK, Phillips AW, Ferri L (2021). Neoadjuvant chemoradiotherapy or chemotherapy alone for oesophageal cancer: population-based cohort study. Br J Surg.

[17] Yang H, Liu H, Chen Y (2018). Neoadjuvant chemoradiotherapy followed by surgery versus surgery alone for locally advanced squamous cell carcinoma of the esophagus (NEOCRTEC5010): a phase III multicenter, randomized, open-label clinical trial. J Clin Oncol.

[18] Sawada G, Niida A, Uchi R (2016). Genomic landscape of esophageal squamous cell carcinoma in a Japanese population. Gastroenterology.

[19] Oh M, McBride A, Yun S (2018). BRCA1 and BRCA2 gene mutations and colorectal cancer risk: systematic review and meta-analysis. J Natl Cancer Inst.

[20] Shenoy S (2019). CDH1 (E-Cadherin) mutation and gastric cancer: genetics, molecular mechanisms and guidelines for management. Cancer Manag Res.

[21] Ko JM, Ning L, Zhao XK (2020). BRCA2 loss-of-function germline mutations are associated with esophageal squamous cell carcinoma risk in Chinese. Int J Cancer.

[22] Yan T, Cui H, Zhou Y (2019). Multi-region sequencing unveils novel actionable targets and spatial heterogeneity in esophageal squamous cell carcinoma. Nat Commun.

